# Development and validation of a nomogram to predict symptomatic intracranial hemorrhage following endovascular treatment in acute ischemic stroke: A single center retrospective, observational study

**DOI:** 10.1097/MD.0000000000042495

**Published:** 2025-05-23

**Authors:** Lvjie Shen, Bintao Jin, Cao Jiang, Jianqiang Yu, Shuxing Li, Yunhui Wang

**Affiliations:** a Emergency Department, Deqing People’s Hospital, Deqing, Zhejiang Province, China; b Department of Neurology, Deqing People’s Hospital, Deqing, Zhejiang Province, China; c Deqing Spine and Spinal Cord Research Center, Deqing, Zhejiang Province, China; d Department of Neurosurgery, Deqing People’s Hospital, Deqing, Zhejiang Province, China.

**Keywords:** acute ischemic stroke, endovascular treatment, nomogram, predictive modeling, symptomatic intracranial hemorrhage

## Abstract

Symptomatic intracranial hemorrhage (SICH) is a severe post-endovascular treatment (EVT) complication of acute ischemic stroke, leading to high morbidity, mortality, and neurological deficits. However, despite these risk factors, there is currently no clinically accepted predictive model to predict SICH risk in EVT patients. This study aimed to identify independent perioperative risk factors for SICH and to provide a new nomogram-based predictive model for large vessel occlusion patients. This study retrospectively examined 127 acute ischemic stroke patients receiving EVT from the First Affiliated Hospital of Wenzhou Medical University. Inclusion criteria included the National Institutes of Health Stroke Scale (NIHSS), Alberta Stroke Program Early CT Score (ASPECTS), and American Society of Interventional and Therapeutic Neuroradiology/Society of Interventional Radiology (ASITN/SIR) collateral scores. Using these predictors, a logistic regression model was used to generate the NIHSS/ASPECTS/ASITN (NAA) nomogram. The predictive ability was measured with the area under the receiver operating characteristic curve and calibration plots. Our data demonstrated that SICH patients had significantly higher baseline NIHSS scores (median: 22.38 vs 15.03; *P* < .001), ASPECTS (median: 5.89 vs 7.69; *P* < .001), and ASITN/SIR scores (median: 1.62 vs 2.69; *P* < .001). The NAA nomogram exhibited an area under the receiver operating characteristic curve value of 0.845 (95% confidence interval: 0.763–0.928), which is superior to the predictive performance. Calibration plots were robust in predicting and observing values. The NAA nomogram is a robust predictor of SICH after EVT that is more accurate than any scoring system. More external validation is needed to make it generalizable to diverse clinical settings.

## 1. Introduction

Intracerebral hemorrhage (ICH) is one of the leading complications of endovascular treatment (EVT) for acute ischemic stroke (AIS), which is either symptomatic (SICH) or asymptomatic, according to the Heidelberg Bleeding Classification.^[1,2]^ SICH can happen during or within 72 hours of the procedure and is highly correlated with adverse outcomes, including increased morbidity and mortality. Retrospective studies of 1122 patients across several centers showed significant increases in the death rates from SICH (odds ratio [OR] 3.53, 95% confidence interval [CI] 2.19–5.68, *P* < .0001).^[3]^ Furthermore, only 8.9% of patients with SICH had good neurological outcomes, based on an modified Rankin Scale score of 0 to 2 at 90 days, as opposed to 51.2% of patients without SICH (*P* < .001).^[4]^

Before the Heidelberg Bleeding Classification, definitions of SICH were based on the European Co-operative Acute Stroke Study-II, the National Institute of Neurological Disorders and Stroke, and the Safe Implementation of Treatment in Stroke-International Stroke Thrombolysis Registry.^[5,6]^ The SICH rates for randomized controlled trials ranged from 3.6% to 9.3%,^[6,7]^ and the highest (9.3%) was seen in the THERAPY trial.^[8]^ On the other hand, SICH was not observed in the SWIFT PRIME, EXTEND-IA, and PISTE trials. Observational studies showed higher rates, ranging from 1.9% to 15.8%.^[9,10]^ Since adherence to the Heidelberg criteria, reported SICH rates increased from 16.0% to 18.6%, reflecting discrepancy in definition and reporting.^[11]^

Several recent studies have identified some key clinical and imaging factors associated with increased risk of SICH following EVT. These include high National Institutes of Health Stroke Scale (NIHSS) scores for severe neurological loss, diabetes, and late start-up.^[12]^ The NIHSS score at baseline is one of the most important variables that relate with clinical outcome.^[13]^ The risk for SICH has also been associated with imaging features, including a low Alberta Stroke Program Early Computed Tomography Score (ASPECTS), minimal infarct core-ischemic penumbra mismatch, very low cerebral blood flow, and low Clot Burden Score (CBS).^[14]^ In addition, low collateral circulation, according to the American Society of Interventional and Therapeutic Neuroradiology/Society of Interventional Radiology (ASITN/SIR) collateral score also has a similar relationship.^[15,16]^ Further operative conditions such as reperfusion injury and device vessel damage can increase the SICH risk.^[17]^

Although the current understanding is well-established, no valid and broadly applied predictive model of SICH following thrombectomy exists. This study sought to quantify independent perioperative risk factors and to develop a novel nomogram-based model for patients with anterior large vessel occlusion (LVO). This model would allow clinicians to use this model to measure SICH risk after direct thrombectomy or bridging therapy, improving patient outcomes.

## 2. Methods

### 2.1. Ethics approval and consent to participate

The Ethics Committee of the First Affiliated Hospital of Wenzhou Medical University (LL2024-K172) approved the study, and informed consent was obtained from all patients.

### 2.2. Patients

All AIS patients with LVO who had undergone EVT at the First Affiliated Hospital of Wenzhou Medical University between November 2017 and March 2019 were enrolled. The therapy was intravenous tissue plasminogen activator in conjunction with EVT or EVT alone. This study included patients presenting to the Emergency Department within 6 hours of symptom onset with baseline computed tomography angiography reporting LVO in the anterior circulation (internal carotid artery anterior cerebral artery A1/A2, middle cerebral artery M1). Those with posterior circulation artery occlusions were excluded.^[18]^ Not included were patients with previous spontaneous intracranial bleeding, which, according to current guidelines, may be harm caused by intravenous alteplase use in those situations. The other exclusion criteria included renal disease (creatinine clearance < 60 mL/min), contrast allergy, hypoglycemia (serum glucose < 2 mmol/L), or uncontrolled hypertension (pretreatment systolic blood pressure ≥ 200 mm Hg). Although patients with high baseline systolic blood pressure or a history of spontaneous intracranial bleeding were excluded, treatment was undertaken with informed consent from the families in light of the increased possibility of complications.

### 2.3. Clinical data and imaging evaluation

Demographic data (age, sex, medical history (smoking, alcohol use), atrial fibrillation, hypertension, diabetes mellitus, initial NIHSS score, dyslipidemia) were gathered during hospitalization. Each patient received a non-contrast computed tomography scan before treatment to rule out intracranial hemorrhage and determine ASPECTS values of early ischemia. On baseline computed tomography angiography, they determined acute occlusions of the anterior circulation and clot features (location and CBS). Pre-EVT digital subtraction angiography for collateralization grade (ASITN/SIR) was performed in the operating room using digital subtraction angiography. ASPECTS, CBS, and ASITN/SIR were measured in a blinded fashion by 2 trained neurointerventionists (ZQ.L. and Q.Y.) and 2 vice chiefs, collectively evaluated more than 500 patients annually. For the sake of objectivity, scores were averaged. In case of disagreement, a third neurointerventionist (M.Z.), the Chief of Neurology at the First Affiliated Hospital of Wenzhou Medical University, was asked to decide.

### 2.4. Treatment process

The EVT procedure was performed on all patients enrolled according to the new protocol for AIS.^[8,18]^ Each patient had a successful thrombectomy with a stent-like retriever (Solitaire FR, Covidien, Irvine, CA). Recanalization success involves a modified Thrombolysis in Cerebral Infarction (mTICI) score of 2b or 3.

### 2.5. Diagnosis of SICH

MR imaging or computed tomography scans were utilized for follow-up within 24 to 72 hours to determine the presence of ICH. SICH was identified and categorized by the Heidelberg Bleeding Classification.^[19]^ SICH was determined by the continued decline in Glasgow Coma Scale scores based on Heidelberg Bleeding Classification scale.

### 2.6. Statistical analysis

All participants were then employed to prepare the prediction model using the statistical package R Project for Statistical Computing and SPSS 22.0 (IBM, Armonk, NY). The variance between the groups with and without SICH was determined by the Mann–Whitney *U* test for continuous variables and the *χ*^2^ test or Fisher exact test for categorical variables.

In the multivariate analysis, predictors that showed at least marginal significance (*P* < .05) were fed into a logistic regression model to generate the nomogram. Collinearity between combinations of variables from the training data were checked, and regression coefficients with standard errors, OR, and two-sided 95% CI for each variable in the model were computed.

The nomogram discriminative power was calculated by measuring the area under the receiver operating characteristic curve (AUC-ROC). The risk prediction model was adjusted in the test group by plotting the observed probability of an adverse outcome based on the total nomogram score against the predicted probability calculated from the nomogram.

## 3. Results

A total of 127 patients with complete data were included in this study to make the nomogram. All 127 patients received Solitaire FR stent-like retrievers (Covidien, Irvine, CA). The average age of these patients was 65.9 ± 13.1 years, and the median baseline NIHSS score was 17.2. Of the 127 patients who signed up, 72 (57.7%) arrived at the Emergency Department within 4.5 hours. Of these, 61 (48%) were candidates for intravenous thrombolysis; only 48 (37.5%) received the treatment. In 61 patients (48%), ICH occurred within 72 hours of EVT. By the Heidelberg Bleeding Classification, 37 patients (29.1%) had experienced SICH.

The patients with SICH had a significantly higher mean initial NIHSS score than those without SICH (median, 22.38 vs 15.03; *P* < .001). Additionally, SICH patients were lower on their pretreatment ASPECTS (median 5.89 vs 7.69; *P* < .001) and CBS scores (median 4.92 vs 6.01; *P* = .005). Also, the ASITN/SIR score was lower in SICH patients than in non-SICH patients (median, 1.62 vs 2.69; *P* < .001). A higher proportion of SICH patients than non-SICH patients had over 3 attempts at a retriever during thrombectomy (32.4%) compared with 7.8% (*P* < .001). Detailed clinical characteristics of all patients are listed in Table 1.

**Table 1:** Clinical characteristics of patients.

Characteristics	Total	SICH		*P*-value
		With (n = 37)	Without (n = 90)	
Age, mean (SD), y	65.9 (13.1)	67.2 (9.9)	65.4 (14.2)	.795
Male sex, n (%)	88 (69.3)	27 (73)	61 (67.8)	.564
Location, MCA, n (%)	89 (70.1)	26 (70.3)	63 (70)	.976
Hypertension, n (%)	70 (55.1)	21 (56.8)	49 (54.4)	.812
DM, n (%)	23 (18.1)	6 (16.2)	17 (18.9)	.722
AF, n (%)	49 (38.6)	13 (35.1)	36 (40.0)	.609
Smoking, n (%)	43 (33.9)	12 (32.4)	31 (34.4)	.828
Alcohol, n (%)	39 (30.7)	10 (27)	29 (32.2)	.564
Pre-anticoagulation, n (%)	20 (15.7)	7 (18.9)	13 (14.4)	.529
Baseline measurements				
Pre-SBP, median (IQR), mm Hg	150.6 (128–175)	151.3 (124–188)	150.3 (129.5–170)	.893
ASPECTS, median (IQR)	7.17 (1.78)	5.89 (1.90)	7.69 (1.43)	<.001*
NIHSS score, median (IQR)	17.2 (11–21)	22.38 (14.5–33.5)	15.03 (10–17.3)	<.001*
CBS, median (SD)	5.698 (2.01)	4.92 (1.83)	6.01 (2.03)	.005*
ASITN/SIR, median (SD)	2.38 (1.05)	1.62 (1.09)	2.69 (0.856)	<.001*
Platelet, mean (IQR), 10^9/L	195.0 (153–231)	181.9 (142–208.5)	200.4 (160.5–237.1)	.130
INR, mean (SD)	1.1 (0.1)	1.1 (0.1)	1.1 (0.2)	.738
LDL, median (IQR), mmol/L	2.4 (0.9)	2.5 (0.8)	2.3 (0.9)	.215
Serum glucose, median (IQR), mmol/L	7.9 (6–9.1)	8.5 (6.4–10.1)	7.6 (5.9–8.4)	.075
Procedure process				
Intravenous thrombolysis, n (%)	48 (37.8)	18 (48.6)	30 (33.3)	.106
OTP, median (IQR), min	304.6 (209–376)	286.1 (204–367)	312.1 (217–380)	.426
PTR, median (IQR), min	86.7 (56–112)	84.2 (54.5–101)	87.7 (54–113.3)	.979
OTR, median (IQR), min	390.5 (303–464)	370.4 (286–455.5)	398.7 (313.8–467)	.324
Passes of retriever > 3, n (%)	19 (15.0)	12 (32.4)	7 (7.8)	<.001*
mTICI, 2b or 3, n (%)	110 (86.6)	32 (86.5)	78 (86.7)	.509

AF = atrial fibrillation, ASITN/SIR = American Society of Interventional and Therapeutic Neuroradiology/Society of Interventional Radiology, ASPECTS = Alberta Stroke Program Early Computed Tomography Score, CBS = Clot Burden Score, DM = diabetes mellitus, INR = international normalized ratio, IQR = interquartile range, LDL = low-density lipoprotein, MCA = M1 or M2 segment of the middle cerebral artery, mTICI = modified Thrombolysis in Cerebral Infarction, NIHSS = National Institute of Health Stroke Scale, OTP = symptom onset to groin puncture time, OTR = symptom onset to recanalizing time, Pre-SBP = systolic blood pressure, PTR = groin puncture to recanalizing time, SICH = symptomatic intracranial hemorrhage.

*P < .05.

In a multivariate analysis, 3 scoring systems were independent predictors of SICH: baseline NIHSS score (OR: 1.1; 95% CI: 1.0–1.1; *P* = .024), pretreatment ASPECT score (OR: 0.6; 95% CI: 0.5–0.8; *P* < .001), and ASITN/SIR collateral score (OR: 0.5; 95% CI: 0.3–0.9; *P* = .017). On the other hand, CBS (OR: 0.9; 95% CI: 0. 7–1.1; *P* = .247) and retriever > 3 passes (OR: 1.3; 95% CI: 0.9–2.0; *P* = .147) were not independent predictors of SICH (Table 2).

**Table 2:** Multivariate analysis of predictors of SICH.

Variables	OR	*P*-value	95% Wald CI	
			Lower	Upper
NIHSS	1.1	.024*	1.0	1.1
ASPECT	0.6	.001*	0.5	0.8
CBS	0.9	.247	0.7	1.1
ASITN	0.5	.017*	0.3	0.9
Passes > 3	1.3	.147	0.9	2.0

ASITN/SIR = American Society of Interventional and Therapeutic Neuroradiology/Society of Interventional Radiology, ASPECTS = Alberta Stroke Program Early Computed Tomography Score, CBS = Clot Burden Score, CI = confidence interval, NIHSS = National Institute of Health Stroke Scale, OR = odds ratio, Passes = Passes of retriever, SICH = symptomatic intracranial hemorrhage.

*P < .05.

**Figure 1.**
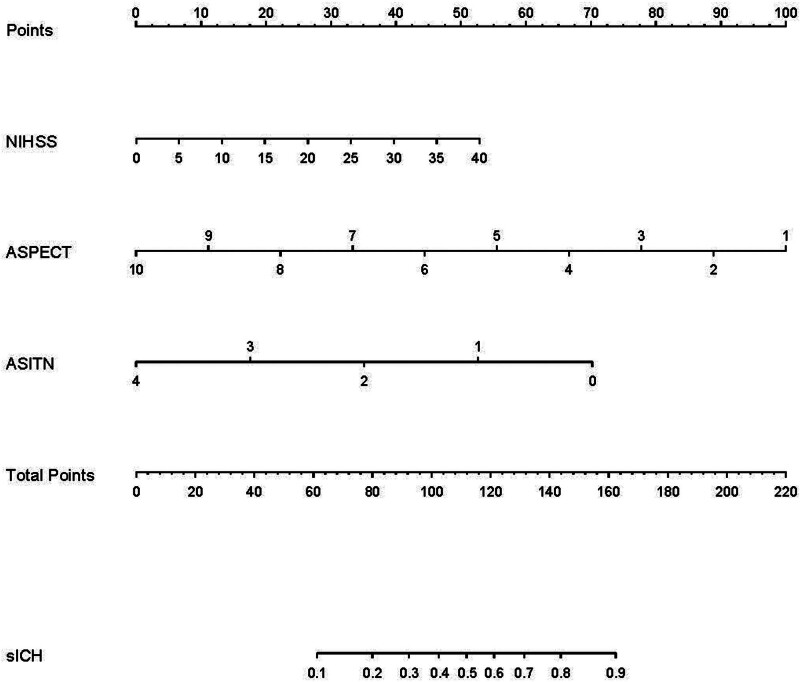
NAA nomogram plots. The initial score of NIHSS was 0 to 40 points, ASPECT was 1 to 10 points, and ASITN was 0 to 4 points and added these to make a total score. The initial score after assessment added together to a total score (0–100 points). Then the total score was converted into a calculated personal SICH risk score after thrombectomy (0–220 points). ASITN = American Society of Interventional and Therapeutic Neuroradiology, ASPECTS = Alberta Stroke Program Early CT Score, NAA = NIHSS/ASPECT/ASITN, NIHSS = the National Institutes of Health Stroke Scale, SICH = symptomatic intracranial hemorrhage.

The 3 pretreatment scoring methods were fed into a logistic regression equation to create the nomogram for estimating the SICH risk. We gave each of the 3 predictors an initial score of 0 to 40 points and added these to make a total score. This aggregate score was converted into a calculated personal SICH risk after thrombectomy, as shown in Figure 1.

**Figure 2.**
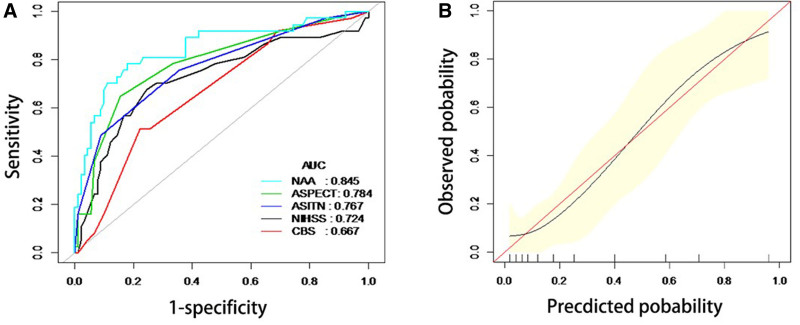
(A) ROC curve for SICH. The NIHSS/ASPECT/ASITN (NAA) nomogram was 0.845, which is highest among the curves. (B) Calibration curve. The NIHSS/ASPECT/ASITN (NAA) nomogram had the excellent correspondence. ASPECTS = Alberta Stroke Program Early Computed Tomography Score, ASITN = American Society of Interventional and Therapeutic Neuroradiology, ROC = receiver operating characteristic curve, NAA = NIHSS/ASPECT/ASITN, NIHSS = the National Institutes of Health Stroke Scale, SICH = symptomatic intracranial hemorrhage.

In 127 patients, the AUC-ROC value of the NIHSS/ASPECT/ASITN (NAA) nomogram was 0.845 (95% CI: 0.7629–0.9275) (Fig. 2A). Internal validation with 1000 bootstraps produced an accuracy of 0.845 (95% CI: 0.7629–0.9273). Furthermore, calibration plots (Fig. 2B) showed excellent correspondence between the predicted NAA nomogram and measured data.

## 4. Discussion

The brain injury caused by ischemia–reperfusion is a multifactorial process: excess free radicals, excitatory amino acid toxicity, intracellular calcium overproduction, and inflammatory mediators.^[20]^ These processes, taken together, all contribute to ischemia–reperfusion-induced neuronal damage and neural dysfunction. What is especially relevant here is the inflammatory response, which is at the heart of this process. After reperfusion, oxygen free radicals induce the production of inflammatory cytokines and chemotactic factors, thereby inducing the expression of adhesion molecules. This process encourages leukocytes to adhere to the endothelial cells in the vascular vessel and cause microvascular occlusion and “no-reflow effect.”^[21]^ Upon accumulation, leukocytes secrete free radicals and proteolytic enzymes, injuring endothelial cells, severing the blood–brain barrier, and leading to a higher incidence of cerebral hemorrhage.^[22]^

In this study, we developed and validated a novel NAA nomogram to predict symptomatic SICH in LVO patients who received direct thrombectomy or bridging therapy. The model unites 3 commonly used scoring algorithms:NIHSS, ASPECTS, and ASITN/SIR collateral scores, that each show a stand-alone relationship with SICH following EVT.

The NIHSS is a well-established tool to evaluate stroke severity and outcome, including the risk of SICH following EVT.^[13]^ In our study, patients with SICH had much higher baseline NIHSS scores than controls (median: 22.38 vs 15.03; *P* < .001), suggesting that it plays a vital role in predicting adverse events. These results are in line with previous research.^[23–25]^ Qian et al NIHSS was found to be an independent risk factor for SICH after thrombectomy (OR: 1.05; 95% CI: 1.01–1.10; *P* = .0263), and their nomogram showed robust predictive activity with an AUC of 0.82.^[23]^ Similarly, Xie et al and Guo et al confirmed the value of NIHSS in predicting SICH after intravenous thrombolysis and its universal application across treatments.^[24,25]^ Zhang et al demonstrate that a decrease in NIHSS scores of 6 or more points within 24 hours strongly predicts favorable outcomes at 3 months, further validating its post-procedural monitoring.^[26]^ The predictive power of NIHSS must lie in its capacity to measure the magnitude of brain dysfunction, which is strongly related to the severity of brain damage and reperfusion injury. Collectively, these results reinvigorate NIHSS as an important diagnostic tool for stroke severity and predictive modeling of SICH and recovery. It is applied in nomograms to boost predictive power and helps ensure accurate risk and treatment recommendations during stroke care.

ASPECTS is a typical quantitative measure for assessing ischemic progression in the MCA territory and helps determine post-EVT outcomes. A lower ASPECTS score is closely related with the high risk of associated with SICH.^[27]^ In our data, patients with SICH had significantly lower ASPECTS scores than those without SICH (median: 5.89 vs 7.69; *P* < .001), and ASPECTS was also independent of SICH (OR: 0.621; 95% CI: 0.467–0.826; *P* = .001). These findings are consistent with the previous report by Zhang et al,^[26]^ who found ASPECTS as a critical predictor for patients following EVT and included it in a nomogram that performed well (AUC 0.795 in the training group and 0.752 in the test group). Similarly, Duan et al reported the predictive ability of ASPECTS in predicting HT following EVT by including it in a nomogram alongside other risk factors with an AUC of 0.797 in the development group and 0.786 in the validation group.^[27]^ ASPECTS thresholds may differ across studies depending on imaging modality and patient selection, but recent studies^[26,28]^ suggest that ASPECTS combined with sophisticated imaging such as computed tomography perfusion or magnetic resonance imaging could further predict hemorrhagic events, maintaining its importance as a key component of risk assessment models.

ASITN/SIR collateral score (0–4) is a standard measure of leptomeningeal collateral circulation, which plays a crucial role in maintaining perfusion in ischemic regions. Lower ASITN/SIR collateral scores are strongly associated with complications such as SICH.^[29]^ In our study, patients with SICH had significantly lower median ASITN/SIR scores than patients without SICH (1.62 vs 2.69; *P* < .001). Any score less than 2 independently was correlated with increased SICH risk (OR: 0.515; 95% CI: 0.299–0.877; *P* = .017). These findings corroborate previous studies showing the impact of poor collateral circulation on the incidence of hemorrhagic complications.^[30,31]^ Lack of adequate collateral flow may prevent full reperfusion, increasing the risk of reperfusion injury and SICH later. Previous studies have also shown similar risk score limits, with below 2 scores most commonly associated with SICH and poor functional status.^[31]^ Nonetheless, our analysis showed slightly lower median ASITN/SIR scores in the SICH group compared with some previous studies, possibly due to differences in imaging technique or scoring system. These associations emphasize the centrality of collateral assessments in advising EVT techniques, which help to determine procedural and therapeutic risks and outcomes.^[16]^

Previous research report about the nomograms combining with biomarkers, clinical imaging and patients’ clinical characteristics such as age, recanalization. These nomograms are effective predictors for the risks of SICH.^[24–26,28]^ However, these nomograms need detailed and varied examination results to make decisions, which maybe accurate but time-consuming. The NAA nomogram for predicting SICH was improved by combining NIHSS/ASPECT/ASITN, which are convenient to access and easy to calculate. This NAA nomogram can be used to roughly estimate the probability of SICH after thrombolectomy at the early screening before the laboratory examinations or imaging evaluations. Especially, in some centers which do not have cerebral perfusion imaging equipment and post-processing software, the NAA nomogram may help the screening a lot. The NAA nomogram in the current study has an enhanced predictive accuracy and discrimination with superior performance, outperforming each scoring system with an AUC-ROC of 0.845 (95% CI: 0.763–0.928). Calibration plots indicated a high level of agreement between predicted and observed outcomes (Fig. 2A and B). The application of NAA nomogram need to be validated by external cohorts.

In this study, the incidence of SICH (29.1%) exceeded rates reported in non-randomized controlled trials (16%). This difference could be attributed to broader treatment criteria, limited operator proficiency during the initial phase of the study, and constraints such as the absence of distal access catheters like Sofia or Navien initially. Over time, improved operator skills and enhanced resources contributed to better outcomes, including a 92% successful recanalization rate (mTICI score 2b or 3) in recent cases.^[32]^ Significantly, cases of SICH more frequently involved multiple retrieval attempts (>3 passes) during thrombectomy (32.4% vs 7.8%; *P* < .001), suggesting a potential link with device-induced vessel injury.

This study has several limitations. First, its retrospective design with a small sample size constrained its robustness, highlighting the need for prospective validation. Second, although the NAA nomogram demonstrated robust calibration and discrimination, external validation in an independent cohort is imperative to confirm its applicability. Third, the current study only included the evaluating scores in the nomagram, which restrict its general application. A more comprehensive model integrating additional biomarkers (e.g., blood-based inflammatory markers, neuroimaging perfusion data) and neuroimaging perfusion data, such as CTP (CBF, CBV, or core infarction volume, ischemic penumbra volume) is needed to potentially refine risk stratification.

## 5. Conclusions

In conclusion, we created and verified a new graphical tool, the NAA nomogram, for forecasting the likelihood of SICH following thrombectomy in individuals with anterior LVO. The NAA demonstrates heightened predictive precision in contrast to singular scoring systems. While the NAA nomogram demonstrates strong predictive performance, its utility would be significantly enhanced through external validation, integration of additional biomarkers, and comparison with existing models. Future research should aim to address these gaps to further improve clinical applicability and patient outcomes.

## Acknowledgments

We express our appreciation to all patients involved in the research.

## Author contributions

**Conceptualization:** Lvjie Shen, Bintao Jin.

**Data curation:** Lvjie Shen, Shuxing Li.

**Formal analysis:** Yunhui Wang.

**Funding acquisition:** Yunhui Wang.

**Investigation:** Cao Jiang, Jianqiang Yu.

**Methodology:** Bintao Jin.

**Project administration:** Yunhui Wang.

**Writing – original draft:** Bintao Jin.

**Writing – review & editing:** Yunhui Wang.
